# Intraocular Foreign Bodies: Clinical Characteristics and Factors Affecting Visual Outcome

**DOI:** 10.1155/2021/9933403

**Published:** 2021-06-18

**Authors:** Yanyan Liang, Shuang Liang, Xiaoli Liu, Danyan Liu, Jialiang Duan

**Affiliations:** ^1^Department of Ophthalmology, Second Hospital of Hebei Medical University, Shijiazhuang, Hebei 050000, China; ^2^Department of Ophthalmology, First Hospital of Hebei Medical University, Shijiazhuang, Hebei 050000, China

## Abstract

**Objective:**

To investigate the clinical characteristics and factors affecting visual outcome in patients with intraocular foreign bodies (IOFBs) and determine the risk factors for the development of endophthalmitis. *Study Design*. A retrospective case-series study design was adopted.

**Subjects:**

In total, 242 patients (242 eyes) who were hospitalized and underwent surgical treatment for IOFB at the Second Hospital of Hebei Medical University between January 1, 2008, and December 31, 2019, were included.

**Methods:**

The demographic data, cause of injury, characteristics of IOFBs, postinjury ocular manifestations, and surgical details of the subjects were collected, and the factors affecting visual outcome and endophthalmitis development were analyzed.

**Results:**

The most common cause of IOFBs was the propulsion of foreign bodies into the eye due to hammering (149 cases, 61.57%), followed by foreign body penetration (57 cases, 23.55%). Most of the subjects were young adult men who sustained injuries in the work environment. Poorer visual outcomes were found in subjects with initial presenting symptoms visual acuity (PVA) < 0.1, largest IOFB diameter ≥ 3 mm, IOFBs located in the posterior segment, wound length > 5 mm, entrance wound length larger than the largest IOFB diameter, concomitant retinal detachment, concomitant vitreous hemorrhage, concomitant endophthalmitis, and concomitant proliferative vitreoretinopathy (PVR). Factors related to the development of endophthalmitis included lens capsule rupture, time of stage 1 repair surgery ≥ 24 h after trauma, removal of IOFBs ≥ 24 h after trauma, and nonadministration of intravitreal antibiotic injection.

**Conclusion:**

Among patients with IOFBs, initial PVA < 0.1, entrance wound length larger than the largest IOFB diameter, concomitant endophthalmitis, and concomitant PVR were risk factors for poor visual outcomes. Lens capsule rupture was a risk factor for endophthalmitis development, and the administration of intravitreal antibiotic injection was a protective factor against endophthalmitis development.

## 1. Introduction

Intraocular foreign bodies (IOFB) refer to foreign objects that penetrate the ocular globe wall and become lodged in the eye. IOFB injuries, which cause varying degrees of damage to ocular tissues, are severe and complex open-globe injuries. In industrialized nations, IOFB injuries are among the most common ophthalmological emergencies causing severe damage to the visual function of young adult men [1]. Previous studies have shown that IOFBs account for 16–41% of all open-globe injuries [1–3], with the majority of IOFBs located in the posterior segment of the eye [4]. IOFB can cause not only mechanical damage to the eyeball but also endophthalmitis and visual function damage, especially IOFB-related endophthalmitis, which is often an emergency. If it is not treated in a timely and effective manner, it may lead to serious consequences and even eyeball removal; therefore, IOFB should be assessed properly. For patients with a poor prognosis and visual acuity and risk factors for endophthalmitis, it is particularly important to conduct a comprehensive assessment for early IOFB diagnosis and development of an effective treatment plan. In the present study, we performed a retrospective analysis of the clinical data of patients with IOFB injuries who underwent surgical treatment at the Ophthalmology Department of the Second Hospital of Hebei Medical University between January 1, 2008, and December 31, 2019, in order to investigate the clinical characteristics of patients with IOFBs, evaluate factors affecting visual outcome, and determine the risk factors for the development of endophthalmitis. The results of this study may serve as guidance for IOFB treatment and visual outcome evaluation in the clinical setting.

## 2. Materials and Methods

Clinical data of patients with IOFB injuries who received medical attention and underwent surgical treatment at the Department of Ophthalmology of the Second Hospital of Hebei Medical University between January 1, 2008, and December 31, 2019, were collected for a retrospective analysis. Patients who were followed up for less than three months were excluded. The follow-up duration was 3–15 months, with an average of 6.02 ± 1.25 months. This study was approved by the hospital ethics committee prior to execution and was conducted in accordance with the Declaration of Helsinki.

**Figure 1:**
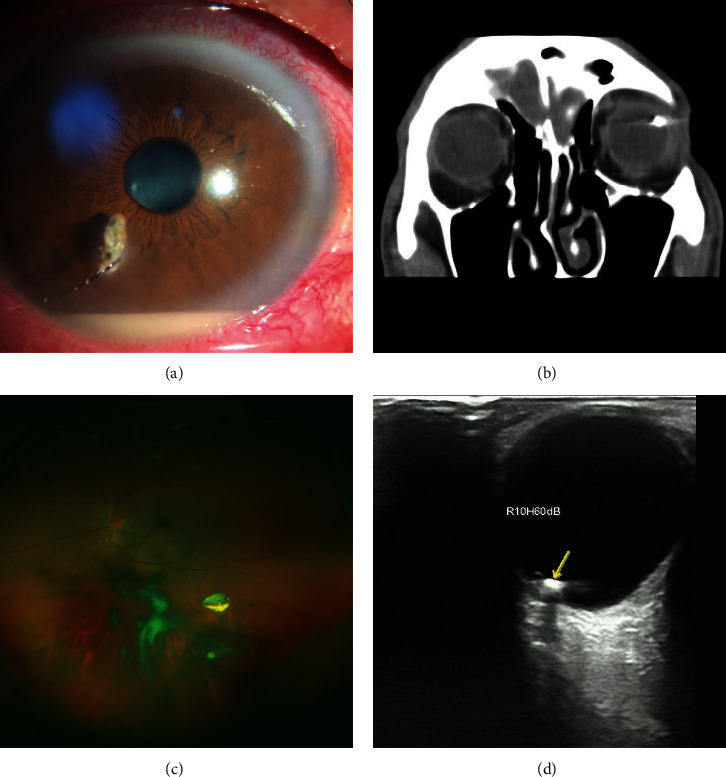
Clinical image of an intraocular foreign body. (a) An intraocular foreign body with an eyelash stuck at the cornea and penetrating the anterior chamber, and the presence of a hypopyon revealed that the patient has concomitant endophthalmitis. (b) Computerized tomography shows a metallic intraocular foreign body. (c) The fundus photo shows a metallic intraocular foreign body in the vitreous cavity with vitreous hemorrhage. (d) B-scan ultrasonography shows an intraocular foreign body with acoustic shadow.

The following data were recorded for all subjects: age, sex, cause of injury, time of injury, ocular manifestations, time of stage I repair surgery, time of IOFB removal, and antibiotic use. Wound sites were classified as follows [5]. (1) Zone I: cornea and corneoscleral limbus; (2) zone II: corneoscleral limbus to a point 5 mm posterior into the sclera; and (3) zone III: posterior to 5 mm from the corneoscleral limbus. The classification for patients with multiple wound sites was based on the most posterior wound. The surgical approach adopted for each patient was determined by taking the nature and location of the foreign bodies and the presence or absence of concomitant endophthalmitis into consideration, and stage I repair surgery was performed at the earliest possible time. The choice of surgical techniques for posterior IOFB includes sclerectomy with extraocular magnets, pars plana vitrectomy with intraocular forceps or intraocular magnets, and C3F8 gas or silicone oil depending on the specific conditions of the retina. For each patient, the decision to administer intravitreal antibiotic injections was made based on the environment where the injury occurred, nature of work performed by the patient, level of cleanliness of the foreign bodies, and the ocular manifestations of the patient during medical consultation (Figure 1).

Subjects were divided into two groups based on the final best-corrected visual acuity (BCVA), namely, the poor visual outcome group (final BCVA < 0.05) and the better visual outcome group (final BCVA ≥ 0.05). The effects of different variables on the final visual outcomes of subjects in the poor visual outcome group and on the development of endophthalmitis in both groups were analyzed using SPSS 20.0. The *χ*^2^ test, rank sum test, or Fisher's exact test was used for univariate analysis, and a logistic regression model was used for multivariate analysis. Differences were considered statistically significant when *P* < 0.05.

## 3. Results and Discussion

### 3.1. Demographic Data

A total of 242 patients (242 eyes) with IOFBs were included in the study, with IOFBs retained in the right eye in 114 patients (47.11%) and in the left eye in 128 patients (52.89%). The subjects consisted of 230 male (95.04%) and 12 female (4.96%) patients with an age range of 4–69 years (average age: 38.47 ± 13.72 years) (Table 1).

### 3.2. Causes of Injury

The most common cause of IOFBs was the propulsion of foreign bodies into the eye due to hammering (149 cases, 61.57%), followed by foreign body penetration (57 cases, 23.55%), propulsion of foreign bodies into the eye due to cutting (18 cases, 7.44%), explosions (10 cases, 4.13%), car accidents (2 cases, 0.83%), and other causes (6 cases, 2.48%). The vast majority of IOFB injuries occurred in the work environment, and detailed medical histories revealed that nearly all the subjects did not use protective eyewear while performing work activities (Table 1).

### 3.3. Ocular Manifestations of Subjects with IOFBs

The most common ocular manifestation was traumatic cataract (191 eyes, 78.93%). Other manifestations included hyphema (40 eyes, 16.53%), vitreous hemorrhage (87 eyes, 35.95%), retinal detachment (60 eyes, 24.79%), and proliferative vitreoretinopathy (PVR) (19 eyes, 7.85%). Only patients with PVR grade >B were included in this study. Based on the PVR grading standard established by the American Retina Association in 1983, among the 19 patients with PVR, 11 eyes were C1, 3 eyes were C2, 4 eyes were C3, and 1 eye was D1, and endophthalmitis (27 eyes, 11.16%). Thirty-eight eyes underwent only one surgery, while 163 eyes underwent two surgeries, and 41 eyes underwent ≥2 surgeries.

### 3.4. The Microbiological Profile of the Patients with Endophthalmitis

Culture was positive in 17 cases and negative in 10 cases. All the 17 culture-positive endophthalmitis were infected by Gram-positive germs (*Staphylococcus epidermis*, *n* = 6; *Staphylococcus aureus*, *n* = 4; and *Streptococcus pneumoniae*; *n* = 3) and Gram-negative germs (*Acinetobacter baumannii*, *n* = 2; and *Escherichia coli*, *n* = 2).

### 3.5. Risk Factors for Poor Visual Outcome

Table 2 provides the results of the univariate analysis. In the poor visual outcome group (final BCVA < 0.05), initial presenting visual acuity (PVA) was <0.1 in 65 eyes (44.52%) and ≥ 0.1 in 10 eyes (10.42%) (*χ*^2^ = 31.497, *P* < 0.001). The proportion of subjects with poor visual outcome was higher in the initial PVA < 0.1 group. The largest IOFB diameter was <3 mm in 19 eyes (21.35%) and ≥3 mm in 56 eyes (36.60%) (*χ*^2^ = 6.121, *P*=0.013), and the proportion of subjects with poor visual outcomes was higher in the largest IOFB diameter ≥ 3 mm group. IOFBs were located in the anterior segment of the eye in 6 eyes (9.68%) and posterior segment in 69 eyes (38.33%) (*χ*^2^ = 17.706, *P* < 0.001), and the proportion of subjects with poor visual outcomes was higher in the group with IOFBs in the posterior segment. Wound length was <3 mm in 26 eyes (20.00%), ≥3 mm and ≤5 mm in 38 eyes (38.38%), and >5 mm in 11 eyes (84.62%), with statistically significant (*P* < 0.001) intergroup differences. Therefore, the proportion of subjects with poor visual outcomes was highest in the group with wound length > 5 mm and lowest in the group with wound length < 3 mm. Entrance wound length was greater than the largest IOFB diameter in 26 eyes (48.15%) and equal to or smaller than the largest IOFB diameter in 49 eyes (26.06%) (*χ*^2^ = 9.567, *P*=0.002), and the proportion of subjects with poor visual outcome was higher in the group with an entrance wound length greater than the largest IOFB diameter. Concomitant retinal detachment was present in 32 eyes (53.33%) and absent in 43 eyes (23.63%) (*χ*^2^ = 18.620, *P* < 0.001), and the proportion of subjects with poor visual outcomes was higher in the group with concomitant retinal detachment. Concomitant vitreous hemorrhage was present in 40 eyes (45.98%) and absent in 35 eyes (22.58%) (*χ*^2^ = 14.262, *P* < 0.001), and the proportion of subjects with poor visual outcomes was higher in the group with concomitant vitreous hemorrhage. Concomitant endophthalmitis was present in 16 eyes (59.26%) and absent in 59 eyes (27.44%) (*χ*^2^ = 11.355, *P* < 0.001), and the proportion of subjects with poor visual outcome was higher in the group with concomitant endophthalmitis. Concomitant PVR was present in 14 eyes (73.68%) and absent in 61 eyes (27.35%), and the proportion of subjects with poor visual outcome was higher in the group with concomitant PVR. Therefore, poorer visual outcome was observed in patients with initial PVA < 0.1, largest IOFB diameter ≥ 3 mm, IOFBs located in the posterior segment of the eye, wound length > 5 mm, entrance wound length greater than the largest IOFB diameter, concomitant retinal detachment, concomitant vitreous hemorrhage, concomitant endophthalmitis, and concomitant PVR. Results of multivariate logistic regression analysis revealed that initial PVA < 0.1, entrance wound length greater than the largest IOFB diameter, concomitant endophthalmitis, and concomitant PVR were risk factors for poor visual outcome (Table 3).

### 3.6. Risk Factors for Endophthalmitis

Univariate analysis showed that lens capsule rupture occurred in 16 eyes (18.18%) and was absent in 11 eyes (7.14%) (*χ*^2^ = 6.885, *P*=0.009) among subjects with endophthalmitis. Preventive intraoperative intravitreal antibiotic injections were administered in 126 subjects, while 101 subjects did not receive injections. To reduce the bias in our results, 15 subjects (15 eyes) with endophthalmitis at the time of medical consultation were excluded. The number of subjects who developed endophthalmitis after initial treatment in the injection and noninjection groups were 1 (0.79%) and 11 (10.89%), respectively (*χ*^2^ = 11.416, *P*=0.001), and the proportion of subjects who developed endophthalmitis was higher in the noninjection group. Among the subjects who developed endophthalmitis, 6 eyes (41.4%) underwent stage I repair surgery at <24 h after trauma and 21 eyes (21.65%) underwent surgery at ≥24 h after trauma, and this difference was statistically significant (*χ*^2^ = 17.981, *P* < 0.001). Therefore, the proportion of subjects with endophthalmitis was higher in the group undergoing stage I repair surgery at ≥24 h after trauma. IOFB removal was performed at <24 h after trauma in 4 eyes (3.33%) and at ≥24 h after trauma in 23 eyes (18.85%), and this difference was statistically significant (*χ*^2^ = 14.699, *P* < 0.001). Therefore, the proportion of subjects with endophthalmitis was higher in the group undergoing IOFB removal at ≥24 h after trauma (Table 4). Multivariate logistic regression analysis (Table 5) showed that lens capsule rupture was a risk factor for the development of endophthalmitis in patients with IOFBs, and intravitreal antibiotic injection was a protective factor against the development of endophthalmitis.

### 3.7. Discussion

IOFBs are a severe ophthalmological emergency and a common cause of blindness [3]. Besides causing mechanical damage to ocular tissues, IOFBs retained in the eye can lead to persistent chemical damage and infection, thereby affecting visual function. In severe cases, blindness or ocular globe atrophy may occur, which may ultimately require enucleation of the affected eye.

In this retrospective case-series study, the data of 242 patients with IOFBs were analyzed. The subjects had an average age of 38.47 ± 13.72 years and included 230 male patients (95.04%), which was consistent with the demographic characteristics of subjects reported in other studies [2, 3]. This is attributed to the fact that young adult men make up the majority of the labor force and have greater exposure to work environments or outdoor activities that pose a high risk of injury. An analysis of the causes of injury revealed that in 149 subjects (61.57%), IOFBs were caused by propulsion of foreign bodies into the eye due to hammering. Previous research has also indicated that hammering is the leading cause of IOFBs mainly due to the lack of eye protection during work involving hammering [2, 3].

#### 3.7.1. Factors Influencing Visual Outcome

Open-globe injury with concomitant IOFB is a complex medical condition, and visual outcomes are affected by a wide variety of factors. In the present study, subjects were divided into two groups using a posttreatment BCVA of 0.05 as the cutoff point, and the risk factors for poor visual outcomes were analyzed in detail.

A retrospective analysis revealed that initial PVA < 0.1 was a risk factor for poor visual outcomes in patients with IOFBs. As initial PVA reflects the degree of damage inflicted by IOFBs on the eye to a certain extent, a poorer initial PVA indicates greater ocular damage, which leads to poorer visual outcome.

Longer wounds were also associated with a poorer final visual acuity in the subjects. This can be attributed to a greater extent of ocular involvement in longer wounds, which leads to a higher possibility of retinal involvement. As the long axis of a foreign body is aligned with its trajectory to reduce resistance during penetration, wound length may provide an indication of the size of the foreign body [6]. However, a large foreign body can cause changes in tissue stress at the wound site, resulting in a tear-like wound that is longer than the foreign body. In addition, fragments that detach from high-speed rotating machinery during mechanical processing carry considerable kinetic energy, which may also create wounds that are larger than the foreign body when the ocular globe is struck horizontally or obliquely. Our results revealed that wound length greater than the largest IOFB diameter was associated with poorer visual outcome in the subjects, making it a risk factor of poor visual outcome. Previous studies found that larger foreign bodies had a greater tendency to cause retinal detachment [7, 8]. In the present study, subjects with an IOFB diameter ≥ 3 mm had poorer visual outcomes than subjects with an IOFB diameter < 3 mm. The entry of larger foreign bodies into the ocular globe causes not only penetrating wounds but also contusions or even tears of differing severity, resulting in wounds that are much larger than the foreign bodies. In addition, larger foreign bodies usually possess greater kinetic energy, which leads to an increased possibility of reaching and damaging the retina [9, 10].

IOFB location also exerted a significant influence on visual outcome [4] as the presence of IOFBs in the posterior segment of the eye was associated with poorer visual outcomes in subjects. This is consistent with the findings of previous studies [2, 3, 10]. IOFBs in the posterior segment have a greater tendency to damage the retina and can cause irreversible vision loss when the macula and papillomacular bundle are involved. However, Anguita et al. adopted the opposite view, concluding that IOFB location was not significantly associated with visual outcome [11]. This view was based on the authors' observation that IOFBs in the posterior segment were mostly located in the vitreous cavity and did not cause retinal damage.

The concomitant occurrence of IOFB injury and posterior segment complications leads to poorer visual outcomes. In this study, 87 subjects developed concomitant vitreous hemorrhage, and of these, 40 (45.98%) patients had a final BCVA < 0.05. This result is consistent with the findings of several studies [1, 12, 13]. However, some studies have also reported the absence of an association between vitreous hemorrhage and visual outcome [14]. Liu et al. concluded that vitreous hemorrhage does not affect visual outcome as it can be completely removed via surgery [15]. However, in our opinion, vitreous hemorrhage usually signifies damage to the posterior segment of the eye. Involvement of the posterior polar retina increases the risk of poor visual outcome; if a vitreous hemorrhage originates from the ciliary body or peripheral retina without involvement of the posterior polar retina, the effect on visual outcome will be smaller after hemorrhage removal.

Once IOFBs cause retinal damage, it is highly likely that retinal detachment will occur, with involvement of the macula usually causing irreversible vision loss [15]. In the present study, 60 subjects exhibited retinal detachment, and among these, 12 subjects developed retinal detachment during follow-up. All of them underwent vitrectomy and received intraocular tamponade treatment. In addition, 32/60 (53.55%) had poor visual outcomes. Previous studies by Guven et al. [16] and Mukkamala et al. [17] have also reported that retinal detachment occurring simultaneously with an IOFB injury or subsequently is a key factor for poor visual outcomes in patients with IOFBs.

PVR was observed in 19 subjects of this study, and of these, 14 (73.68%) exhibited poor visual outcomes. Therefore, concomitant PVR was also a risk factor for poor visual outcomes in patients with IOFBs. PVR refers to the process where proliferative, contractile cellular membranes form on the retinal surface and under the retina, and it is considered the greatest barrier to postoperative retinal reattachment [18]. Results of studies by Szijarto et al. [9] and Wickham et al. [12] also revealed poor visual outcomes in patients with concomitant PVR.

Endophthalmitis is an extremely severe complication of open-globe injury and has an incidence of 6.9–34.78% among patients with IOFBs [2, 19, 20]. Patients who develop endophthalmitis have exceptionally poor visual outcomes. In the present study, 27 subjects developed endophthalmitis, and of these, 16 (59.26%) had a final BCVA < 0.05. Therefore, concomitant endophthalmitis was a risk factor for poor visual outcome among patients with IOFBs.

#### 3.7.2. Factors Unrelated to Visual Outcome

The patients' age was not associated with visual outcomes. We did not observe any association between wound location and visual outcome, which is consistent with the findings reported by Anguita et al. [11]. The nature of the IOFBs and the absence or presence of concomitant traumatic cataract were unrelated to visual outcome also. The timing of repair surgery and IOFB removal was not related to visual outcome. However, the increased risk of endophthalmitis development with delayed stage I repair or IOFB removal cannot be neglected [21, 22].

#### 3.7.3. Factors Affecting Endophthalmitis Development

Previous research on the risk factors for endophthalmitis has mainly focused on open-globe injury [20], while studies on the risk factors for the development of endophthalmitis in patients with IOFBs are relatively scarce. A number of potential risk factors for endophthalmitis have been analyzed in this study.

A total of 88 subjects exhibited lens capsule rupture, and of these, 16 (18.18%) developed endophthalmitis. Among subjects without lens capsule rupture, 11 (7.14%) developed endophthalmitis. With the occurrence of lens capsule rupture, the normal aqueous humor circulation is disrupted, which leads to decreased removal of harmful bacteria in the eye. The ruptured lens may also facilitate bacterial reproduction and cause the entry of bacteria into the vitreous body [23, 24].

Based on the environment where the injury occurred, the nature of work performed by the patient, level of cleanliness of the foreign body, and ocular manifestations of the patient during medical consultation, preventive intravitreal injections of norvancomycin 1 mg/0.1 ml and ceftazidime 1 mg/0.1 ml, which are routine drugs for endophthalmitis prevention [19, 20, 25], were administered to patients at high risk of developing endophthalmitis. Excluding 15 patients with a definite diagnosis of endophthalmitis at the time of consultation, the incidence of endophthalmitis was 10.89% among subjects who did not receive intravitreal antibiotic injections and only 0.79% among those who were administered injections. Therefore, it is evident that intravitreal antibiotic injection is a protective factor against endophthalmitis development. There is a lack of consensus on whether to administer intravitreal injections for the prevention of endophthalmitis [26, 27]. Colyer et al. reported that the incidence of endophthalmitis remained low despite the nonadministration of preventive intravitreal antibiotic injections [27]. However, in our opinion, the IOFB injuries sustained by patients investigated by Colyer et al. were markedly different from typical IOFB injuries. This is because the IOFBs mainly originated from wartime explosions and resulted in extremely high temperatures, which produced sterilizing effects on the IOFBs. Therefore, based on our findings, the administration of preventive intravitreal antibiotic injection for patients with IOFBs is still recommended.

In the present study, 97 subjects received stage I repair surgery at >24 h after trauma, and of these, 21 subjects (21.65%) developed endophthalmitis. However, the incidence of endophthalmitis among subjects undergoing wound closure within 24 h was only 4.14%. Previous research has established that wound suturing or spontaneous wound closure within 24 h after trauma is a protective factor against the development of endophthalmitis in patients with open-globe injury [2]. Our results also indicated that the incidence of endophthalmitis was 3.33% in 120 subjects undergoing IOFB removal within 24 h of trauma, which was considerably lower than that among subjects undergoing IOFB removal > 24 h after trauma (18.85%). However, there is no consensus on whether it is necessary to remove IOFBs as soon as possible [28]. According to some researchers, a delay in IOFB removal does not increase the risk of intraocular infection [2, 23] as the risk of endophthalmitis is elevated by the entry rather than intraocular retention of foreign bodies. The differences between our findings and previously reported results may be caused by the administration of preventive intravitreal antibiotic injections in certain patients during early surgery, which reduced the incidence of endophthalmitis.

There are a few limitations to this study. This was a retrospective study that did not satisfy the design requirements of randomized controlled trials. In addition, the doctors who prescribed the treatment regimens and performed the surgical procedures differed among patients, and this may have resulted in different treatment effects.

## 4. Conclusions

The visual outcome of patients with IOFBs was influenced by multiple factors. Poorer visual outcomes were found in subjects with an initial PVA < 0.1, largest IOFB diameter ≥ 3 mm, IOFBs located in the posterior segment, wound length > 5 mm, wound length greater than the largest IOFB diameter, concomitant retinal detachment, concomitant vitreous hemorrhage, concomitant endophthalmitis, and concomitant PVR. Initial PVA < 0.1, wound length greater than the largest IOFB diameter, concomitant endophthalmitis, and concomitant PVR were the risk factors for poor BCVA. The development of endophthalmitis in patients with IOFBs was also affected by various factors. Results of the univariate analysis revealed that lens capsule rupture, nonadministration of intravitreal injection of antibiotics, time of stage I repair surgery ≥ 24 h after trauma, and time of IOFB removal ≥ 24 h after trauma were associated with a higher risk of endophthalmitis development in the patients. The logistic regression analysis showed that lens capsule rupture was a risk factor for endophthalmitis development, while preventive administration of intravitreal antibiotic injection was a protective factor against endophthalmitis development. Based on these results, we recommend stage I repair surgery for wound closure in a timely manner and the administration of intravitreal antibiotic injection at the earliest possible time to prevent the development of endophthalmitis in patients with IOFBs.

## Figures and Tables

**Table 1:** Demographic data.

Variable		Result
Age (mean, SD, range)		38.47 ± 13.72 (4–69)

Sex (%, number)	Male	95.04% (230)
	Female	4.96% (12)

Affected eye (%, number)	Right	47.11% (114)
	Left	52.89% (128)

Causes of injury (%, number)	Hammering	61.57% (149)
	Penetration	^#1^23.55% (57)
	Cutting	^#2^7.44% (18)
	Explosions	4.13% (10)
	Car accidents	0.83% (2)
	Other causes	2.48% (6)

^#1^Includes intraocular foreign bodies (IOFBs) caused by iron wires, glasses, branches, and other eye-penetrating objects. ^#2^Includes IOFB caused by electric saw, saw machine, and bow saw.

**Table 2:** Univariate analysis of factors affecting the final visual outcome (BCVA < 0.05) of patients with IOFBs.

Factor	BCVA		*X* ^2^	*P*
	≥0.05	＜0.05		
Initial PVA				
** **≥0.1 (*n* = 96)	86 (89.58)	10 (10.42)	31.497	<0.001
** **<0.1 (*n* = 146)	81 (55.48)	65 (44.52)		

Largest IOFB diameter				
** **<3 mm (*n* = 89)	70 (78.65)	19 (21.35)	6.121	0.013
** **≥3 mm (*n* = 153)	97 (63.40)	56 (36.60)		

Nature of IOFBs				
** **Nonmagnetic (*n* = 30)	19 (63.33)	11 (36.67)	0.516	0.473
** **Magnetic (*n* = 212)	148 (69.81)	64 (30.19)		

IOFB location				
** **Anterior segment (*n* = 62)	56 (90.32)	6 (9.68)	17.706	<0.001
** **Posterior segment (*n* = 180)	111 (61.67)	69 (38.33)		

Wound length				
** **<3 mm (*n* = 130)	104 (80.00)	26 (20.00)	—	<0.001^#^
** **3 mm∼5 mm (*n* = 99)	61 (61.62)	38 (38.38)		
** **>5 mm (*n* = 13)	2 (15.38)	11 (84.62)		

Entrance wound length > largest IOFB diameter				
** **No (*n* = 188)	139 (73.94)	49 (26.06)	9.567	0.002
** **Yes (*n* = 54)	28 (51.85)	26 (48.15)		

Wound location				
** **Zone I (*n* = 186)	128 (68.82)	58 (31.18)	2.468	0.291
** **Zone II (*n* = 35)	27 (77.14)	8 (22.86)		
** **Zone III (*n* = 21)	12 (57.14)	9 (42.86)		

Time of stage I repair surgery				
** **<24 h (*n* = 145)	97 (66.90)	48 (33.10)	0.754	0.385
** **≥24 h (*n* = 97)	70 (72.16)	27 (27.84)		

Time of IOFB removal				
** **<24 h (*n* = 120)	86 (71.67)	34 (28.33)	0.787	0.375
** **≥24 h (*n* = 122)	81 (66.39)	41 (33.61)		

Concomitant traumatic cataract				
** **No (*n* = 51)	36 (70.59)	15 (29.41)	0.075	0.784
** **Yes (*n* = 191)	131 (68.59)	60 (31.41)		

Concomitant retinal detachment				
** **No (*n* = 182)	139 (76.37)	43 (23.63)	18.620	<0.001
** **Yes (*n* = 60)	28 (46.67)	32 (53.33)		

Concomitant vitreous hemorrhage				
** **No (*n* = 155)	120 (77.42)	35 (22.58)	14.262	<0.001
** **Yes (*n* = 87)	47 (54.02)	40 (45.98)		

Concomitant endophthalmitis				
** **No (*n* = 215)	156 (72.56)	59 (27.44)	11.355	0.001
** **Yes (*n* = 27)	11 (40.74)	16 (59.26)		

Concomitant PVR				
** **No (*n* = 223)	162 (72.65)	61 (27.35)	17.572	<0.001
** **Yes (*n* = 19)	5 (26.32)	14 (73.68)		

Age				
** **<18 (*n* = 9)	8 (88.89)	1 (11.11)	—	0.136^#^
** **18–60 (*n* = 217)	151 (69.59)	66 (30.41)		
** **>60(*n* = 16)	8 (50.00)	8 (50.00)		

^#^Calculated using Fisher's exact test.

**Table 3:** Logistic regression analysis of factors affecting the final visual outcome (BCVA < 0.05) of patients with IOFBs.

Factor	B	S.E.	Wald	*P*	OR	95% CI for OR	
						Lower	Upper
Initial PVA < 0.1	1.685	0.433	15.152	<0.001	5.394	2.309	12.603
Largest IOFB diameter ≥ 3 mm	0.571	0.453	1.587	0.208	1.769	0.728	4.298
Entrance wound length > largest IOFB diameter	0.927	0.426	4.745	0.029	2.528	1.097	5.822
IOFB location (posterior segment)	0.953	0.534	3.192	0.074	2.594	0.912	7.383
Wound length (<3 mm)					1.000		
Wound length (3–5 mm)	0.228	0.423	0.291	0.590	1.256	0.549	2.875
Wound length (>5 mm)	1.588	0.891	3.177	0.075	4.892	0.854	28.033
Retinal detachment	0.489	0.381	1.645	0.200	1.630	0.772	3.442
Vitreous hemorrhage	−0.046	0.388	0.014	0.905	0.955	0.446	2.043
Endophthalmitis	1.151	0.525	4.806	0.028	3.161	1.13	8.847
PVR	1.393	0.633	4.839	0.028	4.026	1.164	13.924
Constant term	−7.186	1.529	22.090	<0.001			

**Table 4:** Univariate analysis of factors affecting endophthalmitis development.

Factor	Endophthalmitis		*X* ^2^	*P*
	No	Yes		
Lens capsule rupture				
** **No (*n* = 154)	143 (92.86)	11 (7.14)	6.885	0.009
** **Yes (*n* = 88)	72 (81.82)	16 (18.18)		

Nature of IOFBs				
** **Nonmagnetic (*n* = 30)	29 (96.67)	1 (3.33)	1.310	0.252*∗*
** **Magnetic (*n* = 212)	186 (87.74)	26 (12.26)		

Plant-based IOFBs				
** **No (*n* = 238)	211 (88.65)	27 (11.35)		1.000^#^
** **Yes (*n* = 4)	4 (100.00)	0 (0.00)		

IOFB diameter				
** **<3 mm (*n* = 89)	81 (91.01)	8 (8.99)	0.668	0.414
** **≥3 mm (*n* = 153)	134 (87.58)	19 (12.42)		

IOFB location				
** **Anterior segment (*n* = 62)	59 (95.16)	3 (4.84)	3.357	0.067
** **Posterior segment (*n* = 180)	156 (86.67)	24 (13.33)		

Wound length				
** **<3 mm (*n* = 130)	118 (90.77)	12 (9.23)	—	0.488^#^
** **3–5 mm (*n* = 99)	86 (86.87)	13 (13.13)		
** **>5 mm (*n* = 13)	11 (84.62)	2 (15.38)		

Entrance wound length > largest IOFB diameter				
** **No (*n* = 188)	168 (89.36)	20 (10.64)	0.229	0.632
** **Yes (*n* = 54)	47 (87.04)	7 (12.96)		

Wound location				
** **Zone I (*n* = 186)	169 (90.86)	17 (9.14)	—	0.082^#^
** **Zone II (*n* = 35)	30 (85.71)	5 (14.29)		
** **Zone III (*n* = 21)	16 (76.19)	5 (23.81)		

Intraoperative administration of intravitreal antibiotic injection				
** **No (*n* = 101)	91 (90.10)	11 (10.89)	11.416	0.001
** **Yes (*n* = 126)	124 (98.41)	1 (0.79)		

Time of stage I repair surgery				
** **<24 h (*n* = 145)	139 (95.86)	6 (4.14)	17.981	<0.001
** **≥24 h (*n* = 97)	76 (78.35)	21 (21.65)		

Time of IOFB removal				
** **<24 h (*n* = 120)	116 (96.67)	4 (3.33)	14.699	<0.001
** **≥24 h (*n* = 122)	99 (81.15)	23 (18.85)		

^#^Calculated using Fisher's exact test. *∗*Calculated using the *χ*^2^ test with Yates' continuity correction.

**Table 5:** Logistic regression analysis of factors affecting endophthalmitis development.

Factor	B	S.E.	Wald	*P*	OR	95% CI for OR	
						Lower	Upper
Time of stage I repair surgery ≥ 24 h	0.334	1.206	0.077	0.782	1.397	0.131	14.850
Time of IOFB removal ≥ 24 h	2.212	1.510	2.145	0.143	9.131	0.473	26.155
Lens capsule rupture	1.538	0.661	5.414	0.020	4.656	1.274	17.010
Administration of intravitreal antibiotic injection	−2.397	1.111	4.657	0.031	0.091	0.010	0.802
Constant term	−7.270	2.181	11.108	0.001			

## Data Availability

The data used to support the findings of this study are included within the article.
